# In Silico Search for Drug Candidates Targeting the PAX8–PPARγ Fusion Protein in Thyroid Cancer

**DOI:** 10.3390/ijms25105347

**Published:** 2024-05-14

**Authors:** Kaori Sakaguchi, Yoshio Okiyama, Shigenori Tanaka

**Affiliations:** Graduate School of System Informatics, Kobe University, 1-1 Rokkodai, Nada-ku, Kobe 657-8501, Japan

**Keywords:** follicular thyroid neoplasia, PAX8–PPARγ fusion, fusion protein, structure-based drug design, ensemble docking

## Abstract

The PAX8/PPARγ rearrangement, producing the PAX8–PPARγ fusion protein (PPFP), is thought to play an essential role in the oncogenesis of thyroid follicular tumors. To identify PPFP-targeted drug candidates and establish an early standard of care for thyroid tumors, we performed ensemble-docking-based compound screening. Specifically, we investigated the pocket structure that should be adopted to search for a promising ligand compound for the PPFP; the position of the ligand-binding pocket on the PPARγ side of the PPFP is similar to that of PPARγ; however, the shape is slightly different between them due to environmental factors. We developed a method for selecting a PPFP structure with a relevant pocket and high prediction accuracy for ligand binding. This method was validated using PPARγ, whose structure and activity values are known for many compounds. Then, we performed docking calculations to the PPFP for 97 drug or drug-like compounds registered in the DrugBank database with a thiazolidine backbone, which is one of the characteristics of ligands that bind well to PPARγ. Furthermore, the binding affinities of promising ligand candidates were estimated more reliably using the molecular mechanics Poisson–Boltzmann surface area method. Thus, we propose promising drug candidates for the PPFP with a thiazolidine backbone.

## 1. Introduction

Thyroid cancer is a malignant tumor that originates in thyroid cells and can lead to abnormalities in hormone production and nodule formation. Most thyroid cancers contain one of several known driver mutations, such as the V600E substitution in BRAF, RAS mutation, RET gene fusion, or PAX8/PPARγ gene fusion. Various drugs targeting these mutants are currently under development and are expected to become new treatment options. For example, if a patient has a BRAF V600E mutation or an ALK fusion gene, they can receive alectinib in an investigator-initiated clinical trial under patient-directed care [1]. Clinical trials on RET inhibitors for RET gene fusion and medullary thyroid cancer with a positive RET gene mutation are ongoing [2,3]. For unresectable thyroid cancer, the multi-targeted kinase inhibitors sorafenib, lenvatinib, and vandetanib are used as molecular-targeted drugs [4,5,6]. For solid tumors, including NTRK fusion gene-positive thyroid cancer, the ROS1/TRK inhibitor enutrectinib has been approved [7]. However, these therapies simultaneously face limitations of side effects and treatment resistance.

The PAX8/PPARγ rearrangement, producing the PAX8–PPARγ fusion protein (PPFP), is rare in follicular adenomas. It has been reported that follicular adenomas with the PAX8/PPARγ rearrangement are likely to be follicular carcinomas, as these genetic abnormalities are presumed to be involved in the progression from follicular adenoma to follicular carcinoma [8]. The PAX8/PPARγ fusion gene is a translocation between chromosomal regions 2q13 and 3p25, which contain PAX8 and PPARγ, respectively [9]. PAX8 belongs to the paired box family of homeodomain transcription factors, which is expressed in thyroid cells and is required for normal thyroid development. PAX8 mutations are responsible for human congenital hypothyroidism [10]. PPARγ is a member of the PPAR sub-family of nuclear receptors. It forms a heterodimer with retinoid X receptor α (RXRα), another member of the nuclear receptor family, and regulates transcription of target genes in a ligand-dependent manner. PPARγ is a major regulator of adipogenesis and a potent modulator of systemic lipid metabolism and insulin sensitivity [11,12]. It is expressed at very low levels in the normal thyroid. The PPFP is oncogenic, but the exact underlying mechanisms of its activities are not well understood. Focusing on the PPARγ side of the PPFP, several small molecules have been considered as candidates for PPFP-targeted therapeutics [13,14,15]. The PPFP has been described as an inhibitor of PPARγ activity or a transcription factor with proto-oncogene activity [16]; however, in mice, pioglitazone reportedly binds to the PPFP and induces it to behave like PPARγ, resulting in loss of malignancy as thyroid cancer cells differentiate into adipocyte-like cells [9,10,13]. Furthermore, recent clinical trials have demonstrated that pioglitazone induces a significant decrease in thyroglobulin levels and a mild decrease in tumor size [15]. Therefore, it is possible to design a unique anticancer strategy, namely, suppressing malignant transformation by differentiation into noncancerous cells through enhancing the activity of the aberrant PPFP. Despite its importance as an anticancer drug target, there is no effective molecular targeted therapy for the PPFP. The development of small-molecule therapeutics targeting thyroid tumors with the PPFP is expected to establish a standard of care for early stages.

The difficulty in drug discovery of fusion proteins, such as the PPFP, is that their three-dimensional (3D) structure and complete sequence are not yet known; however, recent attempts have emerged to bridge this critical gap [17,18]. Fusion proteins naturally produced by chromosomal translocations, such as oncogenes, have many residues, and other environmental factors, including neighboring proteins, must also be considered. In our previous study [19], we attempted to construct the entire 3D structure of the PPFP using homology modeling based on expected sequential information [10,20,21] and X-ray crystallographic data of the substructures. The PPFP was assumed to be a 901-residue protein consisting of 396 residues of PAX8 on the N-terminal side and 505 residues of PPARγ on the C-terminal side. When 3D modeling the PPFP, the crystal structure of the PPARγ–rosiglitazone complex was used. This PPARγ sequence was the least deficient. The modeled PPFP ligand-binding sites are similar in position but have slightly different shapes. Since the PPFP consists of PAX8 and PPARγ, we also used information on the primary, secondary, and tertiary structures of PPARγ during its modeling. The structure of the refined model was validated using property values and secondary and tertiary structures by a prediction program. Using the modeled structure, we analyzed the fluctuations using molecular dynamics (MD) simulations to validate the structural stability. Furthermore, the molecular mechanics Poisson–Boltzmann surface area (MM/PBSA) study of the binding affinities between PPFP/PPARγ and rosiglitazone revealed different binding free energies for the same binding pocket. We found that fusion of PPARγ and PAX8 distorted the binding pocket structure, which affected the interaction between the ligand-binding domain (LBD) and rosiglitazone. These conclusions were reached within the constraints of the proximity of PAX8 and PPARγ to the flexible missing part being somewhat structured and bound to DNA. Nevertheless, our findings suggest that the ligands that bind the strongest to PPARγ may not strongly bind to the PPFP. These results suggest that there may be optimal compounds for the PPFP that are different from PPARγ, indicating the need for drug discovery targeting this fusion protein.

After we completed the homology modeling of the PPFP, an advanced, artificial-intelligence-based protein prediction methodology called AlphaFold2 appeared [22]. While this new technique has provided a prompt solution to single protein structures given its amino acid sequence, there remain some deficiencies in the prediction accuracy regarding the local structure around the ligand-binding pocket (such as the orientation of side chains) and the complex structure, even if the AlphaFold-Multimer [23] is employed, whose reliability has not yet been confirmed. In this way, we have relied on the homology modeling structure of the PPFP produced in the previous study [19].

Ensemble docking is an efficient and cost-effective technique for compound screening to identify therapeutic drug candidates and has recently received much attention [24,25]. When preparing multiple protein structures for docking screening, it is important to know in advance which protein structure will likely yield the most favorable results, that is, the structure with the highest predictive accuracy. If the active compounds are known, structures that correctly predict their active performance can be employed for screening; however, if not, such a policy cannot be followed. Based on the idea that proteins tend to bind to similar molecules even if they are not truly active compounds, Fukunishi et al. [26] proposed a solution to this problem using the following protocol: a relatively small molecular assembly of existing active compounds can be collected as universal active probes (UAPs), and the protein structure with the pocket with the highest sum of docking scores can be adopted as the screening structure. Because there are no known active compounds for the PPFP except for those targeting PPARγ, we can take advantage of the UAP set for docking structure selection.

In this study, we first discuss the selection of the most suitable PPFP pocket for docking simulations from a large number of PPFP structures generated in the MD simulation of the PPFP apo-form and PPFP–rosiglitazone complex structures. We used PPARγ, for which there is already a large amount of data on activity values and ligands, to verify whether the methodology using the UAP set properly performs and, in that case, what conditions are necessary. Verification included the predictive ability of the docking simulation using the Molecular Operating Environment (MOE) 2020.0901 [27]. To the methodology using the UAP set, we added the condition that the ligand has a thiazolidinedione backbone, which is one of the characteristics of ligands that bind well to PPARγ and its similar structures. Based on these studies, we selected a PPFP structure with a pocket and high activity prediction potential. The selected PPFP structures were docked with 97 compounds with a thiazolidine (TZD) backbone obtained from DrugBank Online (https://go.drugbank.com/ (accessed on 25 March 2024)) [28]. The most favorable drug candidate ligands for the PPFP were also investigated. Finally, we propose the most promising candidate ligands for this fusion protein through re-evaluation using a more reliable MM/PBSA method.

## 2. Results and Discussion

**Figure 1:**
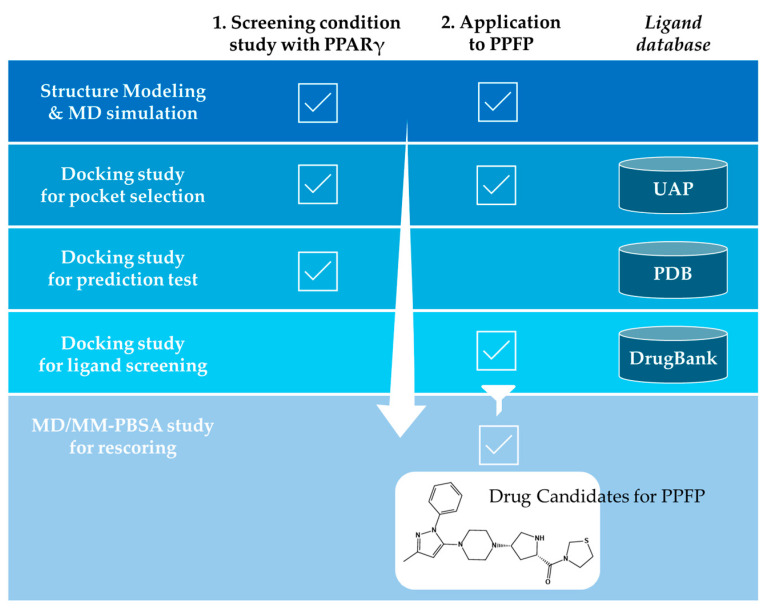
The workflow of the in silico protocol proposed in this work.

As depicted in Figure 1, the goal of this study was to predict promising binders for the PPFP using a database of drugs or drug-like compounds through docking calculations. To this end, it was necessary to select PPFP structures suitable for docking from the MD trajectories of apo-PPFP and PPFP–rosiglitazone complexes modeled in our previous study [19]. Although the position of the PPARγ-derived ligand-binding pocket is the same for all of them, their shapes are slightly different. Here, we first established a protocol to select structures suitable for docking using PPARγ, for which there is abundant knowledge on co-crystal structures and binding affinities with active compounds. We then applied this protocol to the PPFP, for which there is no prior information on its activity or structure, to propose binders as promising target drug candidates.

### 2.1. Protocol for Structure Search for Reliable Docking

To establish a protocol for selecting docking structures with good prediction performance using PPARγ, we first prepared 11 PPARγ structures from two origins, the MD trajectories and the protein data bank (PDB) (Table 1). The former is based on the PPARγ–rosiglitazone complex and apo-PPARγ structures modeled in a previous study [19], giving six representative structures (A01–A06) from the equilibrium states in the 300 K MD simulations. The latter provided five representative crystal structures (A07–A11).

We next docked UAPs consisting of 175 small molecules (Table S1) to the aforementioned 11 PPARγ structures to rank their screening performance by the sum of docking scores (Table 1). The results show that structure ID A11 from the PDB had the best UAP with a docking score sum of −1339.0 kcal/mol, and according to a previous study [26], this structure can be judged as the most suitable for docking screening.

**Figure 2:**
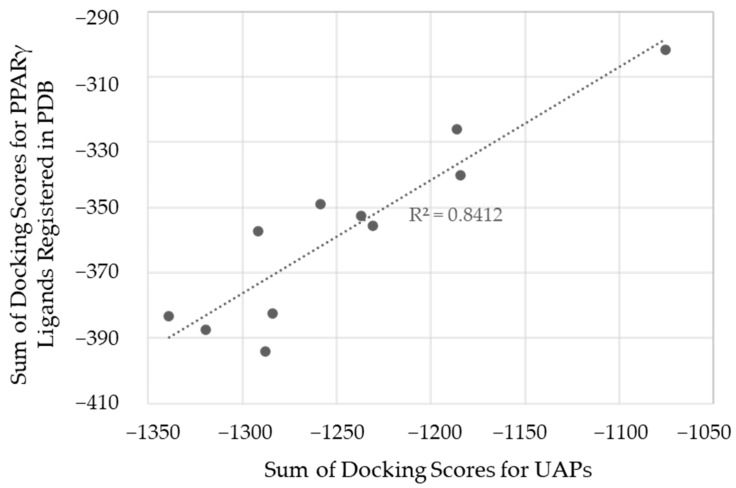
Correlation between the sum of scores obtained by docking the UAP and PDB PPARγ ligands to the 11 PPARγ structures. The UAP is a drug-like small molecule population consisting of 175 compounds. The PDB PPARγ ligands are 41 small molecules with crystal structures and active values. Units are in kcal/mol.

To verify the performance of the UAP protocol on the PPARγ target, we prepared a compound set consisting of 41 PPARγ binders with co-crystal structures registered with PPARγ in the PDB and at least one of the activity values of IC_50_, K_d_, or K_i_ reported in ChEMBL. Then, the sums of the docking scores for the compound set with the 11 PPARγ structures were compared to those of the UAP set to investigate the ability of UAP set for distinguishing the quality of pockets instead of real ligands. Figure 2 shows the correlation between the sum of docking scores for the 11 structures in the UAP- and PDB-derived compound sets. The high correlation coefficient, R^2^ = 0.84, suggests that the UAP protocol would be applicable to PPARγ for the selection of good pockets for screening with high probability. We then suppose that the UAP protocol could also be applied to the selection of appropriate pocket structures in the PPFP, where no experimental data on structure and affinity are available. However, when employing highly diverse UAPs, consideration should be given to keeping the rate of false positives low, which is important in ligand screening. This issue was addressed in this study through combined use of docking score and binding free energy by the MM/PBSA method as a posteriori justification.

To examine the predictive ability of docking for a wide range of PPARγ known active compounds, we performed docking for 11 PPARγ structures of 1696 compounds with activity values (IC_50_, K_d_, K_i_) for PPARγ registered in ChEMBL. IC_50_ is the concentration at which the inhibitory ligand displaces 50% of the substrate. K_d_ is the dissociation constant. K_i_ is the inhibitory constant, defined as the equilibrium concentration of an inhibitory ligand when 50% of the receptor sites are occupied if no competing substrate is present. Since IC_50_ values are calculated based on experimental binding assays and are sensitive to the amount of substrate present, they cannot be compared unless all conditions are equal. On the other hand, K_i_ values can be compared as they are a measure of the direct property of the binding affinity of the inhibitory ligand to its binding partner. The IC_50_ and K_i_ values are related by the Cheng–Prusoff equation [29]. Table 2 shows the correlation coefficient between docking scores and activity values for the corresponding compounds for each activity type and for each PPARγ structure (denoted as “All” in Table 2). Almost no correlation was observed for IC_50_, and weak correlations were observed for K_d_ and K_i_. We then limited our analysis to compounds with TZD and phenylacetic acid (PA) backbones that play important roles in binding to PPARγ; an existing marketed drug, pioglitazone, shares the TZD backbone. Even with these restrictions, we found little improvement in the correlation with IC_50_; however, we obtained noteworthy correlation coefficients (R^2^) with K_i_: for the TZD compounds, 0.482 with the structure ID A03, 0.481 with A05, and 0.491 with A06, and for the PA compounds, greater than 0.5 with many structures. As hypothesized, a binding mode similar to that of TZD, including PA, is expected to improve the prediction performance of K_i_. It is again remarked that K_i_ value can be used to compare the inhibition potency of several compounds for a specific binding partner regardless of the experimental differences in substrate levels.

Since the PPARγ has a large Y-shaped ligand-binding pocket, the chemical space of possible binders is wider [30]. The PPARγ compound group registered in ChEMBL has a wide variation. The selection criteria for the pocket that is expected to give the best docking results are as follows: it can be expected that good results will be obtained if the condition is focused on PPARγ. Among the PPARγ ligands, it seems best to focus on the TZD skeleton characteristic of pioglitazone, which is already a marketed drug. In the case of the PPFP, it is not possible to know the activity value in advance, therefore, it would be beneficial to select the appropriate one based on some molecular characteristics. In terms of the position of the pocket, PPARγ and the PPFP have a pocket in the same position. The dynamics of ligand-binding pockets of protein are then crucial for protein–ligand binding interactions [31]. Therefore, the basic idea of drug discovery is to change the side chain based on the mother skeleton, which is expected to have some activity. First, we focused our discussion on the analogous structure of pioglitazone. Therefore, we propose that the best approach is to have the TZD backbone characteristic of pioglitazone, which is already known to bind well among PPARγ ligands, in addition to previously used methods. Following only the methods of previous studies, the structure selected for PPARγ was PDB-derived A11. The correlation between the docking score and activity value showed that the R^2^ value was not necessarily good. Based on the proposed method, the best structure was A06, which was derived from MD simulations. It had sufficient K_i_ data and a larger R^2^ value than A11. Thus, an even better docking score prediction was expected.

### 2.2. Application to the PPFP

**Figure 3:**
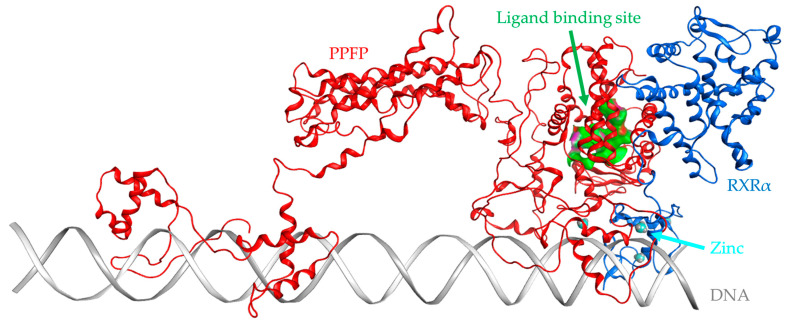
Ribbon drawing of PPFP/RXRα on DNA. Red denotes the PPFP and blue RXRα. The PPARγ used for modeling PPFP is known to form a dimer with RXRα. Zinc ions are shown as balls. The ligand-binding pocket in PPFP is shown in green.

Based on the PPARγ considerations above, a screening protocol that provided the highest predictive accuracy was applied to the PPFP. As in the case of PPARγ, but without any crystal structures, 19 representative structures with structural IDs B01–B19 were extracted from the MD trajectories for the rosiglitazone complex and apo structure in our previous study (Table 3). After evaluation of binding capacities by the UAP set, structure ID B14, showing the highest value of the sum of docking scores (−1303.2 kcal/mol) was selected as the best pocket structure for screening the PPFP (Figure 3).

In this study, we attempted to identify PPFP target drug candidates from the perspective of drug repositioning. The compounds to be screened were obtained from DrugBank [28], an online database of drug and drug candidate compounds and their targets, which includes 9213 available compounds as of November 2022. Based on the scope of the proposed protocol, 97 compounds with TZD backbones were identified (Table S2). Because we focused on comparing the drug candidates with existing drugs, such as pioglitazone and rosiglitazone, candidates with a PA backbone were not considered in this study. The 97 compounds were docked to the PPFP structure with an ID of B14. Table 4 shows the top 10 scoring compounds, along with pioglitazone and rosiglitazone. Pioglitazone and rosiglitazone are widely known PPARγ drugs; however, their binding scores with PPFP were not very high in this study.

**Figure 4:**
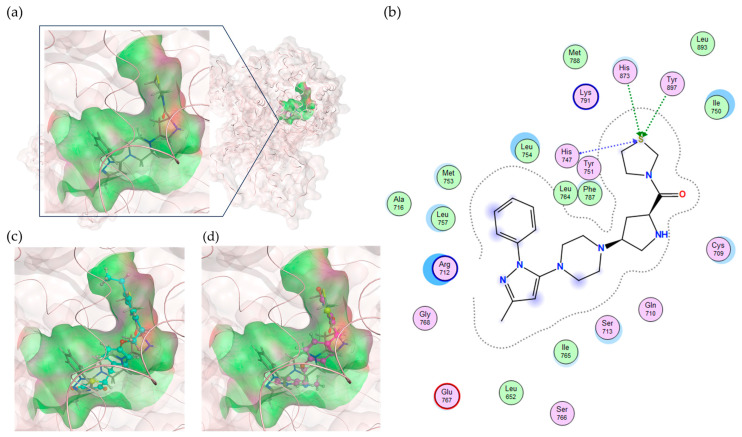
Binding pose of tenegliptin (L72) in PPFP. (**a**) Three-dimensional view, where L72 is represented by stick. (**b**) Two-dimensional view. (**c**) Superposition of L72 (stick) to pioglitazone (cyan) in binding pocket. (**d**) Superposition of L72 (stick) to rosiglitazone (magenta) in binding pocket.

For a more reliable binding-affinity evaluation, multi-sampling MM/PBSA calculations were performed to rescore the 12 docked compounds, including pioglitazone and rosiglitazone. Table 5 shows that teneligliptin (L72), a DDP-4 inhibitor, was the most promising binder for the PPFP. Rosiglitazone (L04) and pioglitazone (L18) had high binding affinities. For ligand candidate L13, the docking score was good; however, the binding free energy was −11.98 kcal/mol, excluding it from the candidate list. Although pioglitazone and rosiglitazone are naturally good candidates, we also determined better binders, such as teneligliptin (see Figure 4), using our computational protocol.

Teneligliptin is already an approved drug for the treatment of type 2 diabetes [32]. Therefore, it would be relatively easy to confirm the efficacy and safety of teneligliptin, such as whether it can be absorbed when administered orally, whether its degradation rate in the small intestine, liver, and blood is sufficiently slow to maintain blood levels, and whether there are any toxicities or side effects. The pharmacokinetics and pharmacodynamics of other identified candidate compounds would be assessed as well since we have employed the strategy of drug repositioning in the present study.

**Figure 5:**
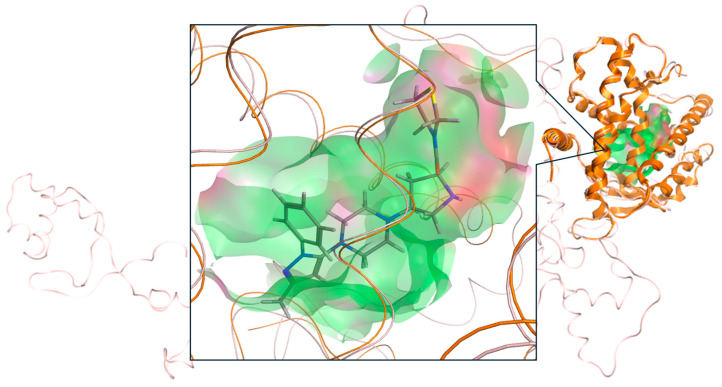
Binding pose of tenegliptin (L72) in PPARγ (PDB ID: 3VJI). The crystal structure of PPARγ (orange) is superimposed on the position of PPARγ in PPFP (pink), and the ligand-binding pocket of PPARγ is shown in green. Superposition of L72 (stick) in the binding pocket is also shown.

Since the crystal structure of the PPFP is still unresolved, it is difficult to make comparisons using the PPFP pocket for the validation of ligand docking pose. It is possible to use the PPARγ pocket, which has the same pocket position but a slightly different shape, and compare the ligand poses by superimposing PPARγ and rosiglitazone, which have a complex crystal structure, with the present results to show that there is no significant discrepancy. We illustrate this comparison in Figure 5.

## 3. Materials and Methods

### 3.1. Protein Structures for Docking

#### 3.1.1. PPARγ Structures

We prepared 11 PPARγ structures categorized into two structure types. Numerous structures were generated by MD simulations during the modeling of the PPARγ apo-body and the PPARγ–rosiglitazone complex structures in our previous study [19]. These were clustered based on the shape of the ligand-binding sites using the density-based spatial clustering of applications with noise (DBSCAN) method of the Amber tool in the MD analysis program. Finally, six structures were selected. On the other hand, the Site Finder application in MOE 2020.0901 [27] was used to detect pocket shapes in 256 PPARγ–ligand complex crystal structures downloaded from the PDB. The features of the pocket shapes were quantified using Sterimol parameters [20,21]. The numerical values were used to cluster the molecules using the k-means method; the Sterimol parameters are a set of vectors describing the steric occupancy of a molecule. They have been extensively used in quantitative structure–activity relationship (QSAR) studies for drug discovery [22,23]. To this end, five structures were selected PDB ID: 1I7I, 2ZK1, 3ADS, 3H0A, and 3VJI. Structures downloaded from the PDB were preprocessed using MOE for docking simulations [27]. Hydrogen atoms were added using Protonate3D. Amber10:EHT was used as the force field for structural optimization, by which the locations of hydrogen atoms were optimized.

#### 3.1.2. PPFP Structures

We created 19 PPFP structures. In our previous study [19], we proposed a homology modeling construction of the entire 3D structure of the PPFP, which is still unresolved, based on X-ray crystal structure data for PPARγ and PAX8. The amino acid sequences of human PAX8 and human PPARγ were retrieved from UniProt [33] (https://www.uniprot.org/ (accessed on 25 March 2024)) with the IDs of Q06710-1 and P37231-1, respectively. To validate these model structures, we analyzed the fluctuations using MD simulations and predicted the physical properties based on the structures and sequences. Many trajectories extracted from these MD simulations were clustered using the DBSCAN method of the AmberTools 14 in the MD analysis program [34]. The parameter radius distance was set to ε = 0.9 and the threshold with neighborhood density was set to minPts = 25. The clustering criterion was the root-mean-square deviation (RMSD) of the residues around the PPFP pocket. The sampled temporal range was between 10 and 100 ns, which was determined to be the equilibrium state, and 18,000 frames were read every five frames. The top clusters, which accounted for more than 1% of all frames, were extracted, and their centroid was used as the representative structure in the equilibrium state. Numerous PPFP–rosiglitazone complex and PPFP apo-body structures, which were previously modeled [19], were generated using MD simulations. These were also clustered based on the shape of the ligand-binding sites. Because the crystal structure of the PPFP was not available, only the MD simulation-derived structures were used. The same procedure as for PPARγ was performed for the 19 prepared PPFP structures.

### 3.2. Compound Sets for Docking

#### 3.2.1. UAPs

Fukunishi et al. [26] proposed the UAP set, which is a collection of relatively small molecules among active compounds against various targets, to evaluate the screening ability of the pocket. This evaluation is based on the idea that similar molecules tend to bind to a pocket, even if it is not the actual active compound of the pocket. Therefore, the pocket with the highest score obtained by docking UAPs to multiple pockets is considered the binding pocket suitable for screening active compounds. In this study, the UAP set consisting of 175 small drug-like compounds registered in the PDB was obtained from MyPresto version 5 (https://www.mypresto5.jp (accessed on 25 March 2024)).

#### 3.2.2. PPARγ Ligands Registered in ChEMBL

One hundred and ninety five (195) PPARγ–ligand complex crystal structures were available from the PDB. One hundred and forty eight (148) PPARγ ligand activity values (IC_50_, K_d_, K_i_) were in ChEMBL. Among them, 63 PPARγ ligands had both crystal structures and activity values. Excluding duplicates, 41 PPARγ ligands were obtained. These 41 PPARγ ligands were docked against 11 structures of PPARγ.

The target-associated bioactive values for PPARγ were extracted from CHEMBL29 (https://www.ebi.ac.uk/chembl/ (accessed on 25 March 2024)) with the target ID CHEMBL235. This resulted in 1696 compounds with SMILES (Table S3).

The computational analysis procedure was the same even when a different type of ligand was docked to PPARγ. The median and −log values for the activity (such as pK_i_ = −logK_i_) were used in the calculation and analysis; K_i_ had 20 small activity values with pKi ≤ 2, which were excluded as outliers (see Figure S1). First, when all IC_50_ values were used (All), there was little correlation; the 1696 PPARγ ligands obtained from ChEMBL included those with large molecular weights. Lipinski’s rule of five [35,36] summarizes the chemical properties of compounds that are likely to become oral drugs and suggests that one of the properties of the drug is a molecular weight of less than 500.

**Figure 6:**
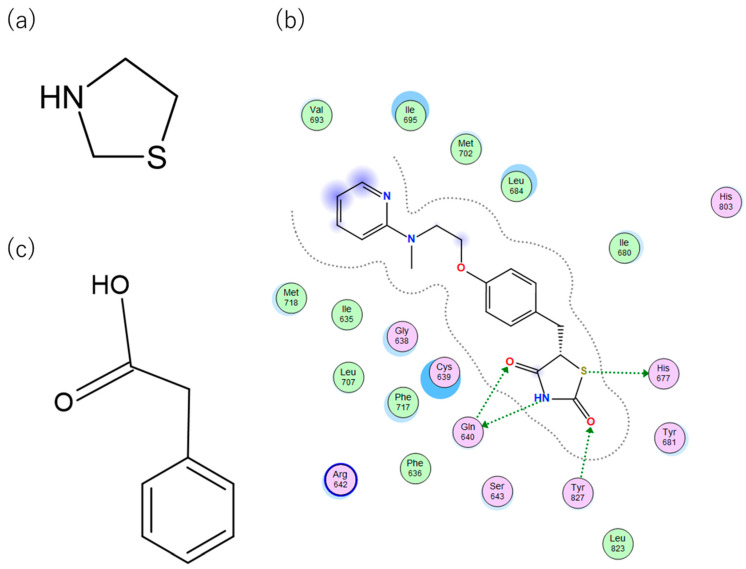
(**a**) Two-dimensional chemical structure of TZD. (**b**) Two-dimensional view of the characterization of the ligand binding sites of PPARγ and pioglitazone. Dotted green arrows indicate interactions between TZD and Gln640, Tyr827, and His677. (**c**) Two-dimensional chemical structure of PA.

TZDs are a class of heterocyclic compounds with thioether and amine groups attached at the first and third positions of the saturated five-membered ring, respectively (Figure 6). The TZD skeleton is characteristic of pioglitazone. Pioglitazone is a ligand for PPARγ, and is already a marketed drug; a two-dimensional view of the ligand binding sites of PPARγ and pioglitazone is displayed in Figure 6. Positional relationships and interactions of the peripheral residues are seen, where hydrogen bonds, ionic bonds, π–π interactions, CH–π interactions, cation–π interactions, halogen bonds, and coordination bonds are anticipated. The TZD backbone plays an important role in the bonding; thus, we extracted 101 compounds with this backbone from the aforementioned 1696 bioactive compounds (Table S3).

PA is another common skeleton of PPARγ binding compounds with a similar binding mode to TZD (Figure 6). To investigate the predicted activity performance of compounds with such similar binding modes, we extracted 31 compounds with a PA backbone from the aforementioned 1696 bioactive compounds of PPARγ (Table S3). Using these experimental values (IC_50_, K_d_, and K_i_), we confirmed that they could be reproduced in the order of increasing activity and correlation. This method was applied to the PPFP. In the case of PPARγ, we established a procedure to predict the superiority of activity values from docking calculations, determining the ligand that binds well to the PPFP. All activity values for PPARγ were used for calculation and analysis and were obtained by evaluating median and −log values. For K_i_, 366 original values were obtained, of which 20 extremely low activity values were excluded as outliers.

#### 3.2.3. TZD Backbone Compounds Registered in the DrugBank Database

As of November 2022, there were 9213 ligands registered in the DrugBank database [28]. The TZD backbone portion is a characteristic structure of pioglitazone and plays an important role in the binding of pioglitazone to the ligand-binding site of PPARγ. Similarly, the TZD backbone is thought to play an important role in the binding to the ligand-binding site of the PPFP. A total of 97 ligands with the TZD skeleton were identified. In this study, we did not consider the case of PA because we wanted to compare the candidates with existing commercial drugs, pioglitazone and rosiglitazone.

### 3.3. Computational Methods

#### 3.3.1. Docking

Docking simulations were performed using MOE 2020.0901 [27]. The final binding free energies and docking scores were evaluated using the London dG and GBVI/WSA dG scoring functions.

#### 3.3.2. MM/PBSA Method

The MM/PBSA approach is an efficient method for evaluating the binding free energy ($∆G_{\mathrm{bind}}$) of protein-ligand complexes [37]. We used the MMPBSA.py module of AmberTools 14 to calculate the binding free energies of 10 ligands considered promising candidates for the PPFP with the PPFP, pioglitazone with the PPFP, and rosiglitazone with the PPFP, and investigated their binding affinities [38]. First, MD simulations were run for 10 ns to investigate the conformational fluctuations at equilibrium in solution (see Figure S2). The protocol for this MD simulation was the same as that previously published [19]. Consequently, 100 structures were extracted from the MD trajectories of the production runs. The scores were reevaluated using the multi-sampling MM/PBSA method [39]. The binding free energies were calculated using the MM/PBSA method to determine the binding affinity of the receptor (PPFP) and ligand (several small molecules of the candidate drugs), where structural fluctuations are taken into account through MD trajectories and some entropic contributions to free energies are approximately incorporated in terms of evaluation of solvent accessible surface area. The difference between the free energy of the complex and those of the ligand and receptor is the binding free energy (Equation (1)):

(1) $$∆G_{\mathrm{bind}}=G_{\mathrm{complex}}-G_{\mathrm{receptor}}-G_{\mathrm{ligand}}.$$

The $∆G_{\mathrm{bind}}$ can also be decomposed into ligand–residue interactions [38]. In this study, the dielectric constant of the solute (protein, internal dielectric constant) was 4.0, while that of the solvent (water, external dielectric constant) was 80.0.

#### 3.3.3. How to Calculate the Correlation Coefficient

The median K_i_ activity values were obtained from experimental data and used for the correlation analysis. The negative logarithm of these values (pK_i_) was calculated and taken on the ordinate. The score values obtained by docking the PPARγ ligands registered in ChEMBL to the prepared structures were taken on the abscissa. Using these variables, the correlation coefficient (R^2^) was calculated. This was performed for each prepared structure. The IC_50_ and K_d_ values were processed in the same way.

## 4. Conclusions

A method for selecting protein structures with pockets suitable for ligand docking from numerous protein structures was developed, and correlations between docking scores of PPARγ and PPARγ ligands and their activity values were examined. Correlations were checked to determine whether they could be reproduced in the order of the experimental activity. The method used in a previous study using UAP is promising. To this method, we added the condition that the PPARγ ligands have a TZD backbone characteristic of the existing drugs pioglitazone and rosiglitazone. In this way, the correlations improved. We applied this method to the PPFP and identified small molecules with a TZD moiety as promising drug compounds. In addition to PPARγ ligands included in existing drugs, compounds with structures similar to TZDs, such as PA, may also be candidates. This suggests the need to search for drug candidates within the PPFP pocket rather than the PPARγ pocket. The top ten compounds reported herein are expected to be potential candidate drugs for the PPFP, for which the ligands are promising.

## Figures and Tables

**Table 1:** Score and ranking of UAP docking to prepared PPARγ structures. The scores are in kcal/mol and UAP is a small-molecule population consisting of 175 drug-like molecules. The sum of the docking scores was obtained by adding all scores obtained for a single structure. Preparation of PPARγ structures and the origin and ID name of each structure.

Score Rank	Sum of Scores	Structure ID	Origin of Structure
1	−1339.0	A11	X-ray crystal structure with PDB ID: 3VJI
2	−1319.3	A09	X-ray crystal structure with PDB ID: 3ADS
3	−1291.9	A06	Centroid of second cluster for MD trajectory of complex structure with rosiglitazone
4	−1288.0	A07	X-ray crystal structure with PDB ID: 1I7I
5	−1283.8	A10	X-ray crystal structure with PDB ID: 3H0A
6	−1258.5	A01	Centroid of first cluster for MD trajectory of apo structure
7	−1236.8	A08	X-ray crystal structure with PDB ID: 2ZK1
8	−1231.1	A05	Centroid of first cluster for MD trajectory of complex structure with rosiglitazone
9	−1186.2	A03	Centroid of third cluster for MD trajectory of apo structure
10	−1184.4	A04	Centroid of fourth cluster for MD trajectory of apo structure
11	−1075.7	A02	Centroid of second cluster for MD trajectory of apo structure

**Table 2:** Correlation coefficient R^2^ between docking scores of 11 prepared PPARγ structures with PPARγ ligands obtained from ChEMBL and their activity values. “All” represents all of the respective activity value data; “TZD” represents compounds with a thiazolidine backbone; “PA” represents compounds with a phenylacetic acid backbone.

Structure ID	IC_50_			K_d_	K_i_		
	All	TZD	PA	All	All	TZD	PA
A01	0.057	0.000	0.0115	0.165	0.176	0.321	0.584
A02	0.052	0.010	0.0019	0.033	0.081	0.201	0.026
A03	0.065	0.000	0.0371	0.376	0.169	0.482	0.500
A04	0.006	0.226	0.1212	0.014	0.091	0.160	0.560
A05	0.067	0.003	0.0543	0.055	0.118	0.481	0.519
A06	0.070	0.000	0.0588	0.378	0.171	0.491	0.571
A07	0.052	0.019	0.0066	0.063	0.052	0.390	0.576
A08	0.050	0.001	0.0553	0.242	0.113	0.243	0.482
A09	0.021	0.002	0.0606	0.103	0.147	0.334	0.513
A10	0.013	0.029	0.0016	0.002	0.134	0.232	0.672
A11	0.049	0.022	0.0809	0.372	0.048	0.309	0.492

**Table 3:** Docking scores and ranking of UAP docking to prepared PPFP structures. The scores are in kcal/mol and UAP is a small-molecule population consisting of 175 drug-like molecules. The sum of the docking scores was obtained by adding all scores obtained for a single structure. Preparation of PPFP structures and the origin and ID name of each structure.

Score Rank	Sum of Scores	Structure ID	Origin of Structure
1	−1303.2	B14	Centroid of 14th cluster for MD trajectory of apo structure
2	−1278.6	B06	Centroid of 6th cluster for MD trajectory of apo structure
3	−1276.8	B02	Centroid of 2nd cluster for MD trajectory of apo structure
4	−1276.0	B16	Centroid of 2nd cluster for MD trajectory of complex structure with rosiglitazone
5	−1271.3	B04	Centroid of 4th cluster for MD trajectory of apo structure
6	−1253.0	B12	Centroid of 12th cluster for MD trajectory of apo structure
7	−1250.8	B10	Centroid of 10th cluster for MD trajectory of apo structure
8	−1242.3	B15	Centroid of 1st cluster for MD trajectory of complex structure with rosiglitazone
9	−1240.9	B08	Centroid of 8th cluster for MD trajectory of apo structure
10	−1236.3	B11	Centroid of 11th cluster for MD trajectory of apo structure
11	−1229.1	B17	Centroid of 3rd cluster for MD trajectory of complex structure with rosiglitazone
12	−1225.7	B18	Centroid of 4th cluster for MD trajectory of complex structure with rosiglitazone
13	−1220.5	B19	Centroid of 5th cluster for MD trajectory of complex structure with rosiglitazone
14	−1219.4	B05	Centroid of 5th cluster for MD trajectory of apo structure
15	−1217.0	B13	Centroid of 13th cluster for MD trajectory of apo structure
16	−1213.6	B09	Centroid of 9th cluster for MD trajectory of apo structure
17	−1213.3	B07	Centroid of 7th cluster for MD trajectory of apo structure
18	−1177.6	B01	Centroid of 1st cluster for MD trajectory of apo structure
19	−1170.1	B03	Centroid of 3rd cluster for MD trajectory of apo structure

**Table 4:** Data on top 10 docking scores, ligand ID names, generic names, and 2D structures.

Score Rank	Docking Score *	Ligand ID	Generic Name	2D
1	−9.837	L62	Lobeglitazone	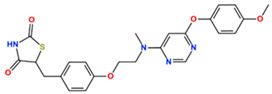
2	−9.387	L03	Piperacillin	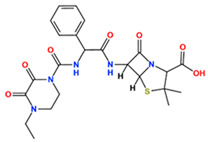
3	−9.346	L21	Bacampicillin	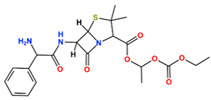
4	−9.248	L96	Ebopiprant	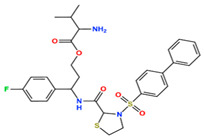
5	−9.162	L29	JE-2147	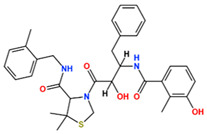
6	−9.133	L72	Teneligliptin	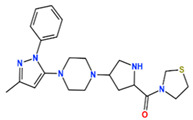
7	−9.119	L87	Talampicillin	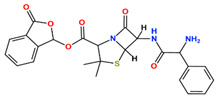
8	−9.080	L13	Mezlocillin	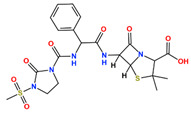
9	−8.931	L23	Pivampicillin	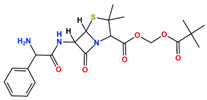
10	−8.693	L58	(5R)-5-(4-{[(2R)-6-HYDROXY-2,5,7,8-TETRAMETHYL-3,4-DIHYDRO-2H-CHROMEN-2-YL]METHOXY}BENZYL)-1,3-THIAZOLIDINE-2,4-DIONE	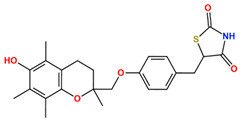
28	−8.009	L18	Pioglitazone	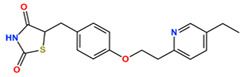
43	−7.643	L04	Rosiglitazone	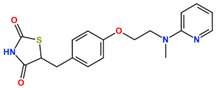

* Score values are in kcal/mol.

**Table 5:** Calculation of MM/PBSA binding free energies for PPFP candidates.

Ligand ID	Delta Total Mean (s.d.)	Score Rank
L72	−25.21 (3.05)	6
L29	−22.82 (4.44)	5
L21	−22.73 (4.49)	3
L03	−21.76 (3.69)	2
L62	−21.15 (3.36)	1
L96	−21.09 (3.49)	4
L04	−20.89 (3.10)	43
L87	−20.32 (3.66)	7
L58	−20.22 (3.25)	10
L23	−17.79 (4.57)	9
L18	−17.22 (2.99)	28
L13	−11.98 (4.40)	8

The unit of binding free energy (Delta Total) is kcal/mol. “s.d.” means the standard deviation.

## Data Availability

The raw data supporting the conclusions of this article will be made available by the authors on request.

## Supplementary Materials

The supporting information can be downloaded at: https://www.mdpi.com/article/10.3390/ijms25105347/s1.
